# Immune Responses of the Critically Endangered Yangtze Finless Porpoises (*Neophocaena asiaeorientalis* ssp. *asiaeorientalis*) to Escalating Anthropogenic Stressors in the Wild and Seminatural Environments

**DOI:** 10.3389/fphys.2019.01594

**Published:** 2020-02-04

**Authors:** Ghulam Nabi, Ying Li, Richard W. McLaughlin, Zhigang Mei, Kexiong Wang, Yujiang Hao, Jinsong Zheng, Ding Wang

**Affiliations:** ^1^The Key Laboratory of Aquatic Biodiversity and Conservation, Institute of Hydrobiology, Chinese Academy of Sciences, Wuhan, China; ^2^University of Chinese Academy of Sciences, Beijing, China; ^3^Key Laboratory of Animal Physiology, Biochemistry and Molecular Biology of Hebei Province, College of Life Science, Hebei Normal University, Shijiazhuang, China; ^4^General Studies, Gateway Technical College, Kenosha, WI, United States

**Keywords:** biodiversity, conservation, habitat, immunity, stressors, Yangtze finless porpoise

## Abstract

Increasing anthropogenic stressors are potential threats to biodiversity conservation and management of Yangtze finless porpoises (YFPs). The objective of this study was to indirectly compare the habitat quality of a natural reserve, Poyang Lake and a seminatural reserve, the Tian-E-Zhou Oxbow (TZO) in terms of anthropogenic stressors by investigating different stress and immunological parameters in the blood of YFPs. Samples from a total of 74 YFPs from the TZO (*n* = 43) and Poyang Lake (*n* = 31) were collected and analyzed. The animals were divided into ontogenetic groups: male calf, female calf, juvenile female, juvenile male, and adult male, and reproductive groups: pregnant female, lactating female, and pregnant plus lactating. The blood from all the animals was analyzed for general stress (HSP14, SOD1, TXN, and FTL), metabolic stress (ACAT2 and THRA), and immunity-related genes (IL12p40, IFNγ, TNFα; IL1α, IL1ra, COX2, CRPL, IL4, and IL8) using qPCR. YFPs living in Poyang Lake showed an increased relative expression pattern for IFNγ, IL1ra, IL4, ACAT2, and CRPL across all the ontogenetic groups with significantly higher expression in adult males. In contrast, YFPs living in the TZO showed a significantly higher expression in 13 of 15 genes analyzed in the male calf group. Across the reproductive states for porpoises living in Poyang Lake, eight of the 15 genes in the pregnant female and three of the 15 genes in the pregnant plus lactating group had a significantly higher expression level. However, in YFPs living in the TZO, eight of the 15 genes showed significantly higher expression in the pregnant and lactating groups. There was significantly a higher expression of most of the genes in porpoises living in the TZO compared to the age-matched groups from porpoises living in Poyang Lake. The exception was the pregnant female group. The higher relative expression of stress and immune genes in the TZO porpoise population compared to porpoises living in Poyang Lake suggests the effects of worsening habitat quality, possibly indicating water pollution and lack of feeding resources.

## Introduction

The world’s aquatic environments are undergoing significant alterations because of anthropogenic activities. Currently, approximately 37% of all cetaceans are at risk of extinction (Davidson et al., 2012). Cetaceans are difficult to study, therefore, the conservation status of approximately 40% of cetacean species remains unknown due to insufficient information (“Data Deficient” species) (Davidson et al., 2012). Compared to other ecosystems, biodiversity in freshwater systems is more at risk (Aloo, 2003; Oren et al., 2010). With increasing human impact, approximately 54% of the world’s freshwater mega fauna species have been listed as threatened (vulnerable, endangered, or critically endangered) (IUCN, 2016). Unfortunately, the consequences of increasing perturbed habitat conditions on marine and freshwater cetacean remain poorly understood (Davidson et al., 2012).

The Yangtze finless porpoise (YFP) (*Neophocaena asiaeorientalis* ssp. *asiaeorientalis*) is a critically endangered freshwater cetacean (Wang et al., 2013). It is native to the Yangtze River, Poyang, and Dongting Lakes of China (Mei et al., 2014). For conservation, some animals were relocated in 1990 into a 21 km long natural *ex situ* reserve, the Tian-E-Zhou Oxbow (TZO) (Wang, 2015). Currently, there are approximately 450 YFPs in Poyang Lake (Mei et al., 2014) and over 60 in the TZO (Wang, 2015). Unfortunately, both populations are exposed to various increasing anthropogenic stressors. For example, the habitat quality of Poyang Lake has been significantly altered by water loss due to prolonged drought (Mei et al., 2015; Zhang et al., 2016). The animals living in the Poyang Lake have also been exposed to extensive environmental stressors such as intensive dredging, shipping, oil spills, and noise. Furthermore, the removal of large numbers of fish and shrimps by sand mining machines, the use of harmful and illegal fishing tools, and the practice of overfishing have injured and killed many YFPs. In addition, the availability, quality, and diversity of prey for YFPs have declined from 1954 to 1990 (Chen et al., 2002; Dong et al., 2006; Wang, 2009; Schelle, 2010; Nabi et al., 2018a). Since 1992, animals in the TZO are exposed to heavy pesticides and agricultural run-offs from the nearby farmlands and poultry wastes (Nabi et al., 2017a, 2018a). The YFPs throughout its lifespan ingest and biomagnification several pollutants in various tissues of their bodies at different rates (Dong et al., 2006). However, during pregnancy and lactation, the rate of biomagnification is higher in fetuses and calves compared to adults (Smith and Reeves, 2000; Dong et al., 2006). Despite pollutant exposure, the YFP population in the TZO has a significantly lower body weight/length ratio compared to that in Poyang Lake, suggesting an inadequate feeding resource (Nabi et al., 2017a).

Except for a few physiological studies (Nabi et al., 2017a, b, 2018a,b), little is known about the pathophysiological effects of environmental stressors on YFPs. Pathologic and physiologic responses to environmental stressors have an essential role in allowing animals to cope with the environment and are largely uncharacterized in cetaceans (Fair et al., 2017). The immune system and multiple immune system proteins work together to cope with environmental stressors and to provide protection for the animal (Morey et al., 2015). Chronic stress exerts an immunosuppressive effect. It suppresses the body’s ability to initiate an efficient and prompt immune reaction, and this increases susceptibility to infectious diseases (Morey et al., 2015). Assessing the immune system and stress responses to environmental stressors in YFPs is essential for monitoring their status and improving management and conservation practices (Christine et al., 2016). Considering the exposure of both the TZO and Poyang Lake YFP populations to several anthropogenic stressors, studies are needed to investigate the animal stress response and the possible immunological effects. Cytokines, secreted by immune cells transmit endocrine, paracrine, and autocrine signals and can serve as crucial biomarkers of the health of the animal (Hofstetter et al., 2017). In view of the environmental differences between YFPs living in the wild and in seminatural reserves, it is therefore essential to develop baseline levels for multiple stress and immune system markers in both populations. As a step toward this goal, we undertook this study to indirectly compare the habitat quality of Poyang Lake and TZO in terms of anthropogenic stressors by investigating different stress and immunological parameters in the blood of YFPs.

## Materials and Methods

### Ethics Statement

The study was approved by the Ministry of Agriculture of the People’s Republic of China. The Research Ethic Committee of the Institute of Hydrobiology, The Chinese Academy of Sciences reviewed and approved the procedure for animal chasing, handling, and blood sampling (NNSFC 31430080). In this study, no surgical intervention, anesthesia, and euthanasia were used. The whole study strictly followed the Chinese law and ethical guidelines for wildlife.

### Study Location

Poyang Lake is the largest freshwater lake in China. It is situated at latitude 28°22′—29°45′ north and longitude 115°47′—116°45′ east in Jiangxi Province. The size of the lake is related to seasonal changes. In winter, its size shrinks to 3,000 km^2^, but in summer, it extends to 4,000 km^2^. This lake is fed by five different rivers including the Xiushui River, Xinjiang River, Ganjiang River, Fuhe River, and Raohe River. The maximum depth of the water is 25.1 m (average depth 8.4 m) (Jing, 2008; Sun et al., 2012; Dong, 2013).

The TZO is an oxbow shape natural *ex situ* reserve established in 1992 by the Chinese Government for the conservation of YFPs. It is in Shishou city, Hubei Province on the north bank of the Yangtze River at E112°31′–112°36′, N29°46′–29°51′. The total length of the reserve is 21 km with a width of 1–2 km (Hao et al., 2009; Wang, 2013). Both in the TZO and the Yangtze River, ecological and environmental conditions are identical (Wang, 2015) except for apparently higher water pollution in the TZO from nearby farmland drainage (Nabi et al., 2017a). Unlike the Poyang Lake, there is no dredging and shipping. Furthermore, the reserve is regularly managed, and the health, abundance, anthropometric, physiological, and molecular indices of YFPs are assessed periodically for research and management purposes.

### Study Design

During a routine health assessment capture and release operation, a total of 79 YFPs were captured from the TZO (*n* = 46) and Poyang Lake (*n* = 33). However, blood samples were only available from 74 individuals. Samples were collected from the YFPs living in the TZO in November 2015 and in March 2015 for the animals living in Poyang Lake. Both populations were classified into various life history categories including male calf, female calf, juvenile female, juvenile male, adult male, pregnant female, lactating female (only in the TZO), and pregnant plus lactating. The body length was used to categorize YFPs into calves, juveniles, and adults (Gao and Zhou, 1993). An ultrasound (LOGIQ Book XP, New York, United States) examination of the reproductive tract and the presence of milk in the mammary glands were used to confirm pregnancy and lactation state, respectively. Data for biochemistry and hematology from these samples have been previously reported by Nabi et al. (2017a). A description of the YFPs used in this study is summarized in Table 1.

**TABLE 1:** Morphological information of Yangtze finless porpoises (*Neophocaena asiaeorientalis* ssp. *asiaeorientalis*).

**Tian-E-Zhou Oxbow**					**Poyang Lake**				
**ID**	**BL (cm)**	**BW (kg)**	**BW/BL**	**Status**	**ID**	**BL (cm)**	**BW (kg)**	**BW/BL**	**Status**
TEZ M26	93.25	19.3	0.206	MC	3,361	101	20.8	0.205	FC
TEZ M03	99	20	0.202	MC	3,310	103	29.8	0.289	FC
TEZ M21	102	22.6	0.221	MC	3,391	105	29.5	0.280	FC
TEZ M23	106.5	20.6	0.193	MC	3,375	108	29.6	0.274	FC
TEZ M24	113	27.9	0.246	JM	3,305	107	31.3	0.292	FC
TEZ M01	111	24.9	0.224	JM	3,349	122	36.8	0.301	JM
TEZ M06	119	27.8	0.233	JM	3,392	124	24	0.193	JM
TEZ M07	125	31.4	0.251	JM	3,377	112	31.8	0.283	JM
TEZ M18	133	35.2	0.264	JM	3,394	129	35.7	0.276	JM
TEZ M19	127	31.5	0.248	JM	3,325	129	37.6	0.291	JM
TEZ M28	125.5	34.3	0.273	JM	PYF3	120	28	0.233	JF
TEZ F11	111	23	0.207	JF	3,323	115	34.7	0.301	JF
TEZ F17	116.5	28.2	0.242	JF	3,341	123	40.3	0.327	JF
TEZ F12	121	29.8	0.246	JF	3,368	125	36.3	0.290	JF
TEZ F04	122	28	0.229	JF	3,337	127	45.7	0.359	JF
TEZ F05	125	38.8	0.310	JF	3,364	138	46.8	0.339	AM
TEZ M22	140.5	35.3	0.251	AM	3,326	144	53.1	0.368	AM
TEZ M04	145	45.3	0.312	AM	3,389	145	49.8	0.343	AM
TEZ M05	141	36.8	0.260	AM	3,302	152	53.8	0.353	AM
TEZ M08	148	40.8	0.275	AM	3,319	153	54.4	0.355	AM
TEZ M12	150.6	44.5	0.295	AM	3,355	159.5	69.8	0.437	AM
TEZ M13	153	47.8	0.312	AM	3,301	154	51.2	0.332	AM
TEZ M14	152.5	46.3	0.303	AM	3,356	160	57.2	0.357	AM
TEZ M10	153.5	50.2	0.327	AM	3,340	134	60.2	0.449	PF
TEZ M17	155.75	46.1	0.295	AM	3,381	138	67.5	0.489	PF
TEZ M09	160	NA	NA	AM	3,327	148	NA	NA	PF
TEZ M27	161	NA	NA	AM	PY F02	135	42.6	0.315	PF
TEZ M15	163.2	52.7	0.322	AM	PY F13	149	50.7	0.340	PF
TEZ M16	163.5	59.3	0.362	AM	PY F08	151	55.9	0.370	PF
TEZ M11	167.5	62.3	0.371	AM	PY F11	157	55.6	0.354	PF
TEZ M25	NA	40.2	NA	AM	3,315	131	42.9	0.327	LF
TEZ F25	139.25	45.8	0.328	PF	3,345	134	58.7	0.438	PL
TEZ F19	143	55.3	0.386	PF	3,329	147	72.1	0.490	PL
TEZ F21	147.5	53.4	0.362	PF					
TEZ F18	149.5	51.1	0.341	PF					
TEZ F06	150.6	51.6	0.342	LF					
TEZ F26	141	45.6	0.323	LF					
TEZ F23	137.5	36.9	0.268	LF					
TEZ F24	141.5	42.5	0.300	LF					
TEZ F16	152.6	49.4	0.323	LF					
TEZ F03	153	39.9	0.260	LF					
TEZ F09	160.5	46.7	0.290	LF					
TEZ F01	137	48.9	0.356	PL					
TEZ F22	141	40.6	0.287	PL					
TEZ F13	146	50	0.342	PL					
TEZ F14	137	40.5	0.295	PL					

*BL, body length; BW, body weight; MC, male calf; FC, female calf; JM, juvenile male; JF, juvenile female; AM, adult male; PF, pregnant female; LF, lactating female; PL, pregnant and lactating female.*

### Animal Chasing, Handling, and Blood Sampling

The YFPs from both Poyang Lake and TZO were captured using “sound chase and net capture” (Hua, 1987). The animals were gently chased by several parallel fishing boats for approximately 15 min. The noise of the fishing boat was 4.5 hp and the speed was less than 10 km/h. The animals were then enclosed in soft spacious fishermen nets, removed from the water, and transported to the medical boat for blood sampling and examination.

A detailed summary of the capture method is described by Hao et al. (2009). During blood sampling, a 10 ml disposable syringe (Gemtier, G/Ø/L: 21/0.7/31 mm, 201502, Shanghai, China) was used to collect approximately 10 ml of blood aseptically from the main vein of the tail within 15 min after the capture. For hematology, 1 ml of blood was transferred to sodium heparinized tubes (Nihon, 161–8560, Tokyo, Japan), 2 ml to non-heparinized tubes for molecular analysis, and the remaining blood was centrifuged (Eppendorf AG, 22332, Hamburg, Germany) for 15 min at 1,500 × *g* for serum biochemistry without the addition of any additives. The serum and 2 ml blood samples were placed in liquid nitrogen for storage. The morphometric measurements, body-weight/body-length ratio, ultrasonography, behavioral observation, and breath frequency were also recorded for each individual porpoise. The total body length was recorded from the tip of the beak to the notch in the fluke, while total body weight was measured in the unit of 0.1 kg.

### Total RNA Extraction and cDNA Synthesis

A commercial Bioteke kit (BioTeke, Wuxi, China) was used according to the manufacturer’s protocol for whole-blood RNA extraction. RNA was then dissolved in 50 μl ribonuclease-free water and frozen at −80°C. The integrity and purity of the RNA extracted were checked by gel electrophoresis and then quantified by a Nanodrop 2000 spectrophotometer before use. cDNA was synthesized using the RevertAid First Strand cDNA Synthesis Kit (Thermo Scientific^TM^, CA, United States) according to the manufacturer’s protocol and procedures.

### Candidate Genes, Primer Design, and qPCR

A total of 15 genes were investigated in both porpoise populations (Table 2). Four genes (HSP14, SOD1, TXN, and FTL) were implicated in a generalized stress response (Cantú-Medellín et al., 2011; Silva-Adaya et al., 2014; Theriot et al., 2016; Garbuz, 2017), and two genes (ACAT2 and THRA) were implicated in metabolic stress (Spitz et al., 2015). The remaining nine genes were implicated as part of an immune response, in which five (IL12p40, IFNγ, TNFα, IL1α, and IL1ra) were proinflammatory cytokines (von der Thusen et al., 2003; Collison and Vignali, 2008; Turner et al., 2014) and two (COX2 and CRPL) were proinflammatory mediators (Lagrand et al., 2002; Gandhi et al., 2017). IL8 is a chemoattractant for leukocytes (von der Thusen et al., 2003), and IL4 is involved in the anti-inflammatory response and also promotes different TH2 responses (Cuneo and Autieri, 2009). In cetaceans, Glyceraldehyde 3-phosphate dehydrogenase (GAPDH) has been reported as one of the best normalizing genes (Spinsanti et al., 2006), and it has been used in gene expression studies for many cetaceans (Beineke et al., 2004; Sharp et al., 2006; Fonfara et al., 2007). Therefore, we selected the GAPDH gene as our house-keeping gene for normalizing candidate gene expression. Primer pairs for the qPCR amplification of eight genes were designed using the software Primer 5 based on the YFP sequences obtained from the GenBank Nucleotide Database. Details about the primers used are listed in Table 2. qPCR products were verified by a combination of base pair size and sequencing. The qPCR was performed with Tian Yi Hui-Yuan master mix (Tian Hui Yuan, Wuhan, China) using an applied Biosystems StepOnePlus^TM^ Real-Time System. The relative concentration of each gene was calculated with the Applied Biosystems Sequence Detection Software v. 1.4 using the equation RQ = 2^(–ΔΔCt)^. Three replicates were used to obtain each average Ct value using the ΔΔCt method.

**TABLE 2:** Detail information of the primers.

**Gene name**	**NCBI accession ID**	**Species**	**Primer sequence (5′–3′)**	**Amplicon Size (bp)**	**References**
GAPDH	XR_003002999.1	YFP	**F:** CACTGGCATGGCTTTCCACG	86	Designed in this study
			**R:** CCTCGTATTTGGCAGGTTTCT		
HSPA14	XM_024738477.1	YFP	**F:** ATTCTGTCCCTCCTGATGAAG	95	Designed in this study
			**R:** AAGAGCGTCCTCCACTAAAAG		
CRPL	XM_024751011.1	YFP	**F:** GACAGATATGCACACAAAGGTC	154	Designed in this study
			**R:** CAGGACATTAGGACTGAAGGTC		
SOD1	XM_024758190.1	YFP	**F:** TCAGTGTCTTGTTTCAGTGGT	89	Designed in this study
			**R:** AGGCTTGAGAACTGAAGAAAT		
THRA	XM_024744289.1	YFP	**F:** ACACCCTGACACTGAGCGGG	91	Designed in this study
			**R:** AGTTCAAAGATGGCATCGGAGA		
ACAT2	XM_024747965.1	YFP	**F:** TCACTTGGTTCACTTGAGGACA	107	Designed in this study
			**R:** TGCCCATGTGATAGTTGTGAA		
FTL	XM_024768849.1	YFP	**F:** AGTGGGGTAAAACTCAGGACGC	180	Designed in this study
			**R:** GGTGGTCACCCATCTTCTTGAT		
TXN	XM_024757292.1	YFP	**F:** ATCAAGCCTTTCTTTCATTCTC	95	Designed in this study
			**R:** CACTCTGAAGCAACATCCTGAC		
IL8	DT660217	Bottlenose	**F:** TGTCACTGCAAGCCTTATTATGC		Mancia et al., 2008
			**R:** GTGAATTTTTGCTGTTTTGAGAAAGA		
TNFα		Harbor Porpoise	**F:** GGCTGAACACATATGCCAAC	111	Müller et al., 2013
			**R:** TGAAGAGGACCTGGGAGTAGA		
IL12p40	EU638319	Bottlenose	**F:** CAGACCAGAGCGATGAGGTCTTG	184	Sitt et al., 2008
			**R:** GGGCTCTTTCTGGTCCTTTAAGATA		
COX2	EU638321	Bottlenose	**F:** GGGAGGAAAGAGCTTCCTGATTCAA	188	Sitt et al., 2008
			**R:** GTCCACCCCATGGCTCTGTCC		
IL1α	AB028215.1	Bottlenose	**F:** CAGCTTCCAGAGCAACATGA	129	Hofstetter et al., 2017
			**R:** TTTAATGCAGCAGCCATGAG		
IL1RA	AB038268.1	Bottlenose	**F:** TGTGGCAAAATGGAAAACAA	108	Hofstetter et al., 2017
			**R:** CCCTTCCAGAAAGGACATCA		
IL4	AB020732.1	Bottlenose	**F:** TCTCACCTCCCAACTGATCC	125	Hofstetter et al., 2017
			**R:** TTGCTGTGAGGATGTTCAGC		
IFNγ	AB022044.2	Bottlenose	**F:** GCGCAAAGCCATAAGTGAAC	103	Hofstetter et al., 2017
			**R:** TCTCTGGCCTCGAAACAGAT		

### Statistical Analysis

All data are expressed as means ± SEM using Graph Pad Prism, version 7.04 (Graph Pad Software Inc., San Diego, CA, United States). One-way ANOVA followed by a *post hoc* test (Tukey test) was used to measure relative variations in gene expression across the age groups (calves, juveniles, adults) and reproductive groups (pregnant, lactating, pregnant plus lactating). An unpaired Student’s *t* test was used in the reproductive groups of Poyang Lake population to measure differences in the relative gene expression between pregnant and pregnant plus lactating females. Similarly, an unpaired Student’s *t* test was used to compare one group from one population to its respective group from another population. Normality was assessed using the Shapiro–Wilk test. The alpha (α) set at ≤0.05 indicates a statistically significant difference.

## Results

### Relative Gene Expression Across Age Groups

**FIGURE 1:**
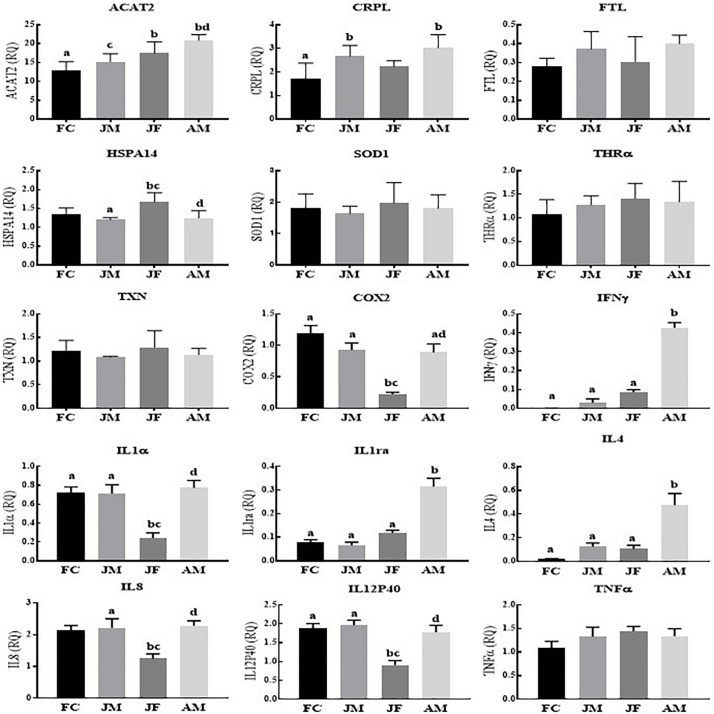
Yangtze finless porpoises (YFPs) living in Poyang Lake across the age groups. Each parameter followed by an alternate letter was significantly different at *P* ≤ 0.05. FC, Female calves; JM, Juvenile males; JF, Juvenile females; AM, Adult males.

In Poyang Lake YFPs, we observed significantly lower relative expression levels of COX2, IL1α, IL8, IL12p40 and significantly higher expression levels of HSPA14 in juvenile female compared to the other age groups (Figure 1). However, the relative expression levels of IFNγ, IL1ra, IL4, ACAT2, and CRPL were statistically significantly higher in the adult male across the age groups. The female calf and juvenile male groups showed significantly higher expressions of COX2, IL1α, and IL12p40 compared to the juvenile females, while IL8 was only significantly higher in the juvenile males *versus* juvenile females. No statistical differences were detected for the expression levels of TNFα, FTL, SOD1, THRα, and TXN (Figure 1).

**FIGURE 2:**
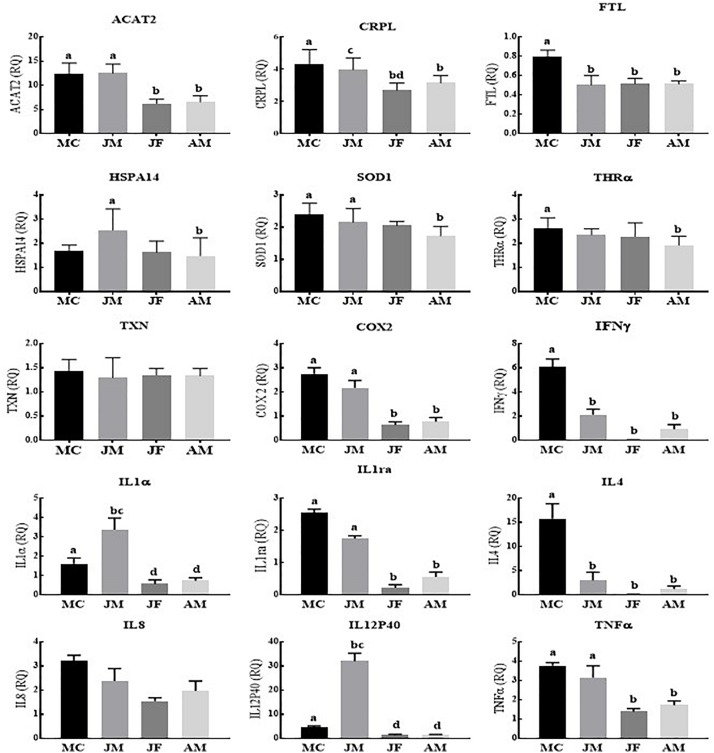
Yangtze finless porpoises (YFPs) living in the Tian-E-Zhou Oxbow across the age groups. Each parameter followed by an alternate letter was significantly different at *P* ≤ 0.05. MC, Male calves; JM, Juvenile males; JF, Juvenile females; AM, Adult males.

In contrast to Poyang Lake, YFPs living in the TZO showed a statistically significantly higher expression of most of the genes (13/15) in male calves across the age groups. Similarly, the expressions of nine genes were significantly higher in juvenile males across the age groups, while no difference was observed in IL8 and TXN expression (Figure 2).

### Relative Gene Expression Across Reproductive States

**FIGURE 3:**
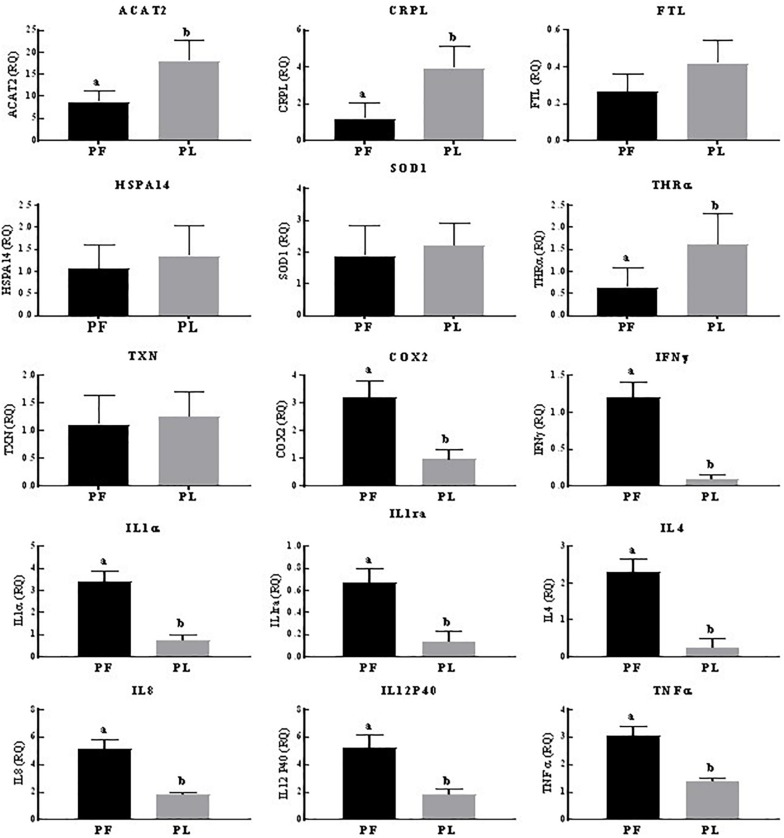
Yangtze finless porpoises (YFPs) living in Poyang Lake across the reproductive status. Each parameter followed by an alternate letter was significantly different at *P* ≤ 0.05. PF, Pregnant female; PL, Pregnant plus lactating female.

**FIGURE 4:**
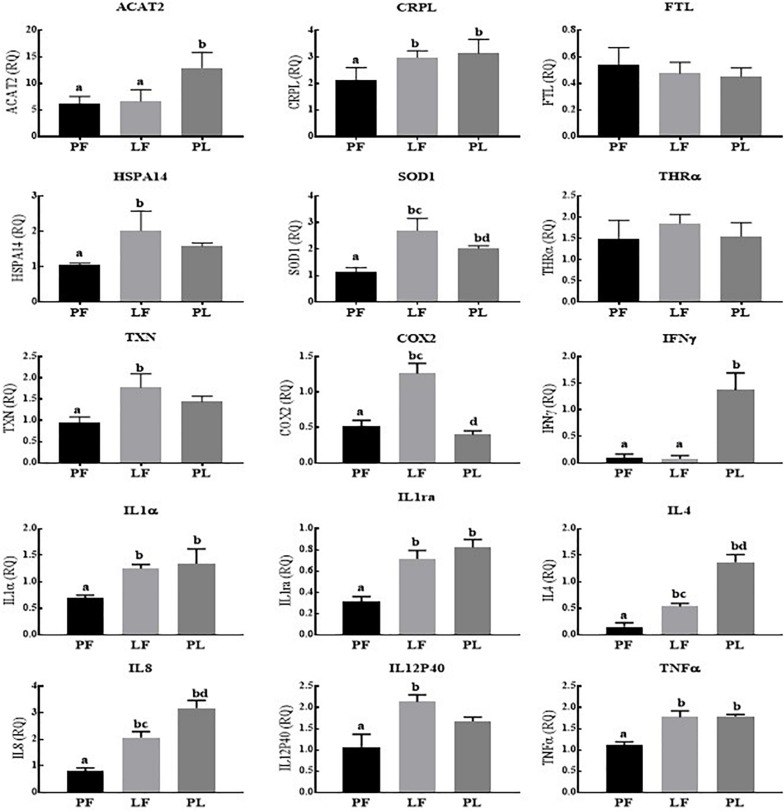
Yangtze finless porpoises (YFPs) living in the Tian-E-Zhou Oxbow across the reproductive status. Each parameter followed by an alternate letter was significantly different at *P* ≤ 0.05. PF, Pregnant female; LF, Lactating female; PL, Pregnant plus lactating female.

Across the reproductive states in porpoises living in Poyang Lake, 8/15 genes (COX2, IL1α, IL1ra, IL4, IL8, IL12P40, TNFα, and IFNγ) were expressed in the pregnant vs. pregnant plus lactating females at statistically significantly higher levels. However, the expression of ACAT2, CRPL, and THRA was significantly higher in the pregnant plus lactating vs. pregnant females. No statistically significant difference was observed for the FTL, HSPA14, SOD1, and TXN expression levels (Figure 3). In porpoises living in the TZO, four genes (ACAT2, IFNγ, IL4, and IL8) in the pregnant plus lactating group were significantly higher than in both the pregnant group and lactating group. Four genes (CRPL, IL1α, IL1ra, and TNFα) in both the lactating group and pregnant plus lactating group were significantly higher than in the pregnant group. Similarly, three genes (SOD1, COX2, and IL12p40) were significantly higher in the lactating group compared to both the pregnant group and pregnant plus lactating group, while two genes (HSPA14 and TXN) in the lactating group vs. the pregnant group were significantly higher. However, no significant difference was detected for FTL and THRα levels across the reproductive states (Figure 4).

### Comparison of Relative Gene Expression in Matched Groups Between TZO and Poyang Lake YFPs Populations

#### Calves

In porpoises living in the TZO, the expression levels of 11/15 genes in calves were significantly higher compared to calves living in Poyang Lake. However, no statistically significant difference was observed in ACAT2, HSPA14, SOD1, and TXN levels between the two populations (Table 3).

**TABLE 3:** Comparisons of qPCR result in matched groups from both populations.

**Gene**	**Calve**	**JM**	**JF**	**AM**	**PF**	**PL**
ACAT2	12.38a±1.34	12.54 ± 2.46	6.26 ± 2.43	6.59 ± 2.34	6.12 ± 2.54	12.96 ± 3.48
	12.81b±2.56	15.23 ± 1.54*	17.53 ± 3.54***	20.89 ± 3.34****	8.76 ± 2.92	18.05 ± 2.58
CRPL	4.3a±1.65*	3.97 ± 1.2*	2.70 ± 1.95	3.16 ± 1.3	1.11 ± 0.65	3.14 ± 0.87
	1.72b±0.98	2.67 ± 0.6	2.24 ± 1.77	3.02 ± 1.5	1.20 ± 0.87	3.95 ± 1.17
FTL	0.8a±0.162**	0.50 ± 0.28	0.51 ± 0.13	0.51 ± 0.16*	0.53 ± 0.21*	0.45 ± 0.12
	0.27b±0.13	0.37 ± 0.20	0.30 ± 0.25	0.4 ± 0.146	0.26 ± 0.09	0.42 ± 0.22
HSPA14	1.69a±0.66	2.55 ± 1.25*	1.62 ± 0.29	1.48 ± 0.93	1.04 ± 0.67	1.58 ± 0.09
	1.36b±0.50	1.2 ± 0.68	1.68 ± 0.96	1.25 ± 0.44	1.06 ± 0.54	1.34 ± 0.68
SOD1	2.40a±0.64	2.15 ± 1.46	2.04 ± 0.95	1.73 ± 0.99	1.15 ± 0.54	2.02 ± 0.50
	1.80b±0.43	1.63 ± 0.92	1.98 ± 1.00	1.80 ± 0.73	1.89 ± 0.64*	2.20 ± 0.70
THRα	2.60a±1.20*	2.35 ± 0.64*	2.28 ± 0.64*	1.92 ± 0.64*	1.5 ± 0.64**	1.55 ± 0.77
	1.07b±0.76	1.27 ± 0.75	1.41 ± 0.41	1.34 ± 0.43	0.65 ± 0.35	1.61 ± 0.70
TXN	1.43a±0.49	1.29 ± 0.80	1.35 ± 0.16	1.33 ± 0.53	0.95 ± 0.55	1.44 ± 0.13
	1.22b±0.47	1.08 ± 0.56	1.29 ± 0.72	1.13 ± 0.37	1.10 ± 0.54	1.25 ± 0.45
COX2	2.76a±0.43***	2.17 ± 0.75**	0.64 ± 0.25*	0.77 ± 0.63	0.51 ± 0.17	0.39 ± 0.09
	1.18b±0.29	0.92 ± 0.24	0.22 ± 0.05	0.89 ± 0.37	3.23 ± 1.49**	0.98 ± 0.30*
IFNγ	6.09a±1.12****	2.12 ± 1.22**	0.02 ± 0.03	0.92 ± 0.70*	0.08 ± 0.08	1.37 ± 0.55*
	0.00b±0.00	0.02 ± 0.04	0.08 ± 0.02*	0.42 ± 0.07	1.2 ± 0.55**	0.09 ± 0.06
IL1α	1.61a±0.52**	3.38 ± 1.34**	0.57 ± 0.21*	0.74 ± 0.20	0.70 ± 0.08	1.34 ± 0.47
	0.71b±0.14	0.71 ± 0.20	0.24 ± 0.09	0.77 ± 0.21	3.40 ± 1.16***	0.76 ± 0.32
IL1RA	2.55a±0.18****	1.75 ± 0.19****	0.22 ± 0.07*	0.55 ± 0.28*	0.31 ± 0.08	0.82 ± 0.12**
	0.07b±0.02	0.06 ± 0.02	0.11 ± 0.01	0.31 ± 0.09	0.67 ± 0.33*	0.13 ± 0.12
IL4	15.79a±5.36***	2.99 ± 0.40****	0.08 ± 0.05	1.23 ± 0.80**	0.15 ± 0.15	1.36 ± 0.24*
	0.02b±0.00	0.12 ± 0.06	0.10 ± 0.02	0.47 ± 0.27	2.32 ± 0.90***	0.24 ± 0.35
IL8	3.21a±0.39**	2.38 ± 1.14	1.54 ± 0.27	1.97 ± 0.76	0.82 ± 0.20	3.18 ± 0.49*
	2.13b±0.34	2.21 ± 0.63	1.24 ± 0.26	2.28 ± 0.43	5.14 ± 1.80***	1.89 ± 0.16
IL12P40	4.56a±0.98***	32.05 ± 7.87****	1.30 ± 0.39	1.77 ± 0.52	1.07 ± 0.59	1.67 ± 0.16
	1.87b±0.29	1.96 ± 0.30	0.90 ± 0.20	1.37 ± 0.80	5.28 ± 2.22**	1.85 ± 0.21
TNFα	3.76a±0.27****	3.14 ± 1.35*	1.39 ± 0.29	1.71 ± 0.81	1.12 ± 0.13	1.79 ± 0.07*
	1.08b±0.32	1.33 ± 0.44	1.44 ± 0.17	1.34 ± 0.44	3.05 ± 0.90**	1.42 ± 0.14

(^*a*^) = TZO, (^*b*^) = Poyang Lake. JM, Juvenile male; JF, Juvenile female; AM, Adult male; PF, Pregnant female; PL, Pregnant plus lactating female. The asterisk (*) for each gene in each group represent significantly high expression at *P ≤ 0.05, **P ≤ 0.01, ***P ≤ 0.001, and ****P ≤ 0.0001.

#### Juvenile Male

Similar to calves living in the TZO, most of the genes (10/15) were statistically significantly higher compared to the juvenile males living in Poyang Lake. Only ACAT2 levels were statistically significantly higher in the Poyang Lake YFPs, while no difference was observed in the IL8, FTL, SOD1, and TXN levels between the two populations (Table 3).

#### Juvenile Female

Unlike calves and juvenile males, most of the gene (9/15) expression levels showed no statistically significant differences. The relative expressions of COX2, IL1α, IL1ra, and THRA were statistically significantly higher in porpoises living in the TZO. However, IFNγ and ACAT2 levels were statistically significantly higher in juvenile females living in Poyang Lake (Table 3).

#### Adult Male

Similar to juvenile females, most of the gene (9/15) expression levels showed no statistically significant differences between the two populations. Only the expression level of ACAT2 in porpoises living in Poyang Lake and five genes (IFNγ, IL1ra, IL4, FTL, and THRA) of YFPs living in the TZO were statistically significantly higher (Table 3).

#### Pregnant Female

Interestingly, most of the gene (9/15) expression levels were statistically significantly higher in YFPs living in Poyang Lake. No significant differences were observed in the expression levels of ACAT2, CRPL, HSPA14, and TXN. Two genes (FTL and THRA) were highly expressed in the TZO population (Table 3).

#### Pregnant Plus Lactating

In this group, only the expression levels of 6/15 genes were statistically significantly high. The expression levels of COX2 in porpoises living in Poyang Lake and IFNγ, IL1ra, IL4, IL8, and TNFα levels in porpoises living in the TZO were statistically significantly high (Table 3).

#### Body-Weight/Body-Length

**FIGURE 5:**
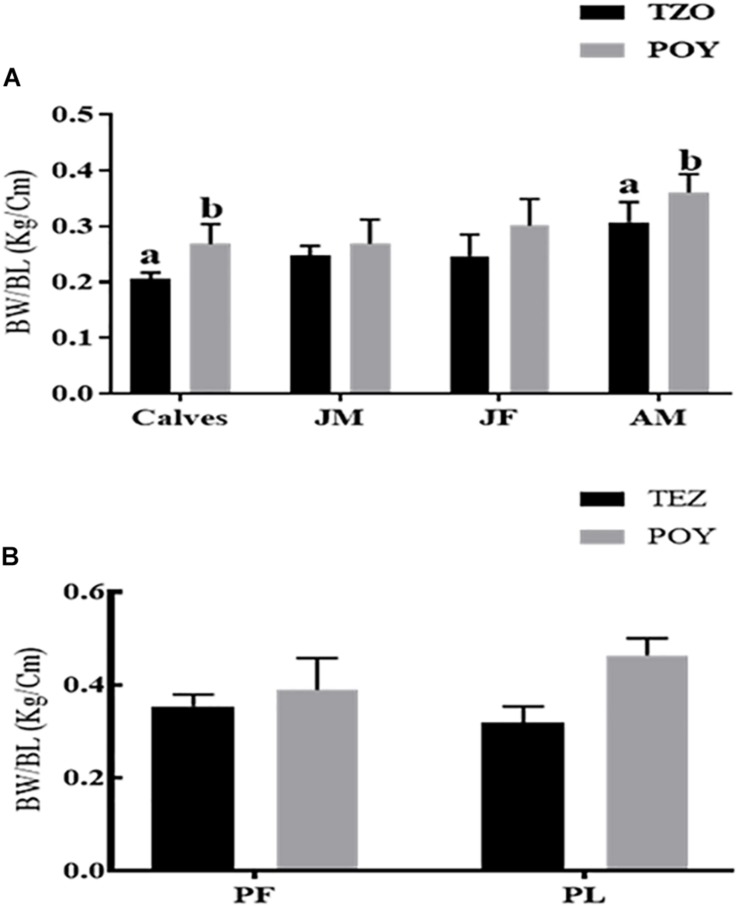
**(A)** Across the age groups and **(B)** across the reproductive status. Overall comparison of the body-weight/body-length between the two populations. The body-weight/body-length in each group followed by an alternate letter was significantly different at *P* ≤ 0.05. JM, Juvenile males; JF, Juvenile females; AM, Adult males; PF, Pregnant female; PL, Pregnant plus lactating female.

The body-weight/body-length ratios of calves and adult males in the TZO were statistically significantly lower compared to the animals living in Poyang Lake. In other groups across the age groups (juvenile male and juvenile female) and reproductive states (pregnant female, pregnant and lactating female), body-weight/body-length was not statistically significantly different. However, the overall trend showed lowered body-weight/body-length in the TZO population (Figure 5).

## Discussion

In this study, we used qPCR to investigate stress, metabolism, and immunity-related relative gene expression levels in YFPs living in the wild compared to a seminatural reserve. This study could help us to understand the individual health responses and physiological impact resulting from the increasing anthropogenic activities. Samples were collected from the two porpoise populations during different seasons; therefore, the possible effect of seasonal variations should be considered in the interpretation of results. We reported significantly high expression levels of stress-related genes (FTL and HSPA14) in YFPs living in the TZO, indicating exposure to a potential stressor in their environment. A hallmark of a stressed organism is the high expression of heat shock genes which function as molecular chaperones for maintaining proteostasis (Garbuz, 2017). The heat shock response in the entire animal kingdom is highly conserved against extreme proteotoxic insults such as oxidative stress, toxins, heavy metals, pathogens, hypoxia, heat, and other harmful conditions, suggesting their importance for survival in a stressful environment (Åkerfelt et al., 2010; Garbuz, 2017). The water in the TZO is more polluted than in Poyang Lake. Pollutants like pesticides and poultry excrement have been entering the TZO for over 20 years. These chemicals can negatively affect the liver profile and other physiological processes in YFPs (Acevedo-Whitehouse et al., 2018). Similarly, a significantly high expression level of FTL in the YFPs living in the TZO also indicates an immunomodulatory response (Zarjou et al., 2019). FTL is highly conserved among species and is involved in iron mineralization, nucleation, and long-term iron storage (Barbeito et al., 2009; Lee et al., 2009), although oxidative stress can release ferrous iron (Fe^2+^) from ferritin, further increasing the iron load in the body (Theriot et al., 2016). However, in a previous study, we did not report any significant difference in the Fe^2+^ concentration between the TZO and Poyang Lake populations (Nabi et al., 2018a). We did not observe a significant variation in the relative expression levels of stress-related genes across the ontogenetic and reproductive states in YFPs living in Poyang Lake. However, in YFPs living in the TZO, the expression levels of stress-related genes were significantly higher at a younger age, and for adult females, expression levels were highest in lactating animals, suggesting that age and reproductive status modulate the response to anthropogenic stressors. The state of pregnancy and lactation could not only affect the response but could also be physiologically challenging in itself (Maestripieri et al., 2008; Luijten et al., 2014; East et al., 2015).

Thyroid hormones (T_3_ and T_4_) and thyroid hormone receptors (THRα and THRβ) have an essential role in the regulation of cetacean metabolism. In cetaceans, diet restriction reduces circulating T_3_ levels but upregulates the expression of both THRα and THRβ levels (Martinez et al., 2013; Spitz et al., 2015). This high level of empty THR (without T_3_) further activates the generally negative regulation of the gene coding for Thyroid Stimulating Hormone (TSH) which reduces T_3_ levels (Oppenheimer and Samuels, 1983). The significant increase in the expression level of THRα, the lower BW/BL, additionally with an abrupt increase in the population (Wang, 2015), reduction in the serum level of T_3_ (Nabi et al., 2018b), and an increase in the fishing activities in the TZO population suggest a reduction in the availability of food resources. The significantly lowered body-weight/body-length and hypercholesterolemia observed in the TZO vs. Poyang Lake YFPs (Nabi et al., 2017a) could also suggest nutritional stress-induced cholesterol mobilization from the adipose tissue (Savendahl and Underwood, 1999; Sokolovic et al., 2010). ACAT2 is an essential metabolic enzyme mainly found in the liver and intestine that catalyzes the formation of cholesteryl esters. In fasting mammals, the expression of ACAT2 is decreased. However, refeeding the animals increases the expression levels (Caimari et al., 2010). In our study, the significantly lowered expression of ACAT2 in YFPs living in the TZO (Table 3), the decreased expression levels in younger compared to older animals (Figure 2), and the increasing expression levels in younger compared to older animals living in Poyang Lake (Figure 1) also reflect the inadequate nutritional resources present in the TZO.

The concentrations of cytokines synthesized by peripheral blood mononuclear cells (PBMCs) provide information on systemic inflammatory trends (Hofstetter et al., 2017). In cetaceans, analysis of cytokines is a relatively new field. Only six studies in cetacean have linked contaminant exposure to cytokine levels (Desforges et al., 2016). Similar to our study, several studies have reported a stress-induced increase in the levels of proinflammatory cytokines (Kiecolt-Glaser et al., 2003; Segerstrom and Miller, 2004; Glaser and Kiecolt-Glaser, 2005; Calcagni and Elenkov, 2006; Webster and Glaser, 2008; Tian et al., 2014). The secretion of proinflammatory cytokines is beneficial in the inflammatory response if secreted in proper amounts, otherwise higher secretion levels can be toxic (Arango and Descoteaux, 2014). Similar to YFPs living in the TZO, other cetaceans exposed to various chemical pollutants (MeHg, Hg, and PCB) both *in vivo* and *in vitro* have increased expression levels of IL4 and IL1 (Kakuschke et al., 2006; Das et al., 2008; Routti et al., 2010; Brown et al., 2014). IL4 is a pleiotropic immunomodulatory Th2 cytokine (Lee et al., 2010). Instead of having anti-inflammatory activity (Rocken et al., 1996), several studies summarized by Lee et al. (2010) have considered IL4 as a proinflammatory cytokine. It synergistically increases the expression levels of TNFα, IL1, and IL1ra (Orino et al., 1992; Lee et al., 2010). The pyrogenic cytokines (IL1 and TNFα) are the first to be secreted in response to pathogens, and their significantly high expression levels in porpoises living in the TZO suggest the stimulation of the acute phase of the immune response to stress and infection (Beutler, 1999; Arango and Descoteaux, 2014). These pyrogenic cytokines further stimulate the synthesis of CRPL and other mediators as observed in the TZO population (Beutler, 1999; Griffin et al., 2012). We also observed significantly high expression levels of IFNγ, IL12, IL8, and COX2 in the TZO population suggesting they may play a role in chronic stress, infectious diseases, and autoimmune pathologies (Shahzad et al., 2010; Sozzani et al., 2014). Chronic stress is associated with high levels of IL8 (Shahzad et al., 2010). It has a role in neutrophil recruitment and in the chemotactic migration and activation of lymphocytes, monocytes, eosinophils, and basophils at the site of inflammation (Turner et al., 2014). IL12 is critical in the defense against intracellular bacteria, viruses, and parasites. It synergizes with other proinflammatory cytokines, including TNFα, in stimulating IFNγ production (Wang et al., 2000), mostly in T-cells and natural killer cells (NK) (Øvergård et al., 2012). The IFNγ further activates macrophages, enhancing phagocytosis and regulating the transcription of hundreds of genes having various immunoregulatory functions in both adaptive and innate immunity (Øvergård et al., 2012). Oxidative stress increases the production of COX2. It is dramatically upregulated by inflammation and can cause tissue damage by producing prostaglandins. Furthermore, proinflammatory cytokines, such as IL1 and TNFα, also positively regulate COX2 expression levels (Lu and Wahl, 2005; Onodera et al., 2015). The high expression level of COX2 in Poyang Lake pregnant females could possibly be related to physical stimuli (vessel trafficking) and hypoxia (Kang et al., 2007; Perera and Herbstman, 2011; Nabi et al., 2018b). However, the cause of hyperimmune responsiveness needs further investigation.

Young animals are more sensitive to stress (Brydges et al., 2012; Luijten et al., 2014) as observed in the TZO YFPs. Unlike adult animals, young animals have a relatively immature immune system which develops with the passage of time and with exposure to several environmental challenges (Simon et al., 2015). In our study, we observed relatively high expression levels of immune system genes in young animals living in the TZO. Both malnutrition and infection are linked. Malnutrition in healthy individuals with a low body-mass index can increase the expression levels of various cytokines (Takele et al., 2016). Both pregnancy and lactating states are energetically the costliest (Scantlebury et al., 2000; Kastelein et al., 2002) and therefore requires increased food intake (East et al., 2015). In the TZO population, the significantly high expression of THRα during pregnancy could reflect reduced feeding resources (Martinez et al., 2013; Spitz et al., 2015), although lactation is even more energy demanding than the pregnant state (East et al., 2015). However, we did not observe a significantly high expression of THRα in lactating animals living in the TZO. This needs further investigation since feeding frequency and extent of parental care could play a role in THRα expression levels. When food resources are insufficient for both self-maintenance and lactation, investments in immune system processes are reduced which can lead to infections as was observed in the TZO population (East et al., 2015).

## Conclusion and Future Recommendations

In summary, our findings indirectly suggest a worsening habitat quality of the TZO both in terms of pollution and feeding resources. Younger animals, especially, are more affected in the TZO compared to animals living in Poyang Lake. The opposite trends we observed for different gene expression levels in both populations across the ontogenetic and reproductive stages could be related to the nature of the different kinds of environmental stressors. The populations of YFPs living in the TZO has increased, however, their persistent exposure to pesticides and declining feeding resources require urgent attention and proper management. Improving feeding resources, regulating the number of porpoises so as not to exceed the carrying capacity of the oxbow, managing poultry and sewage waste, and replacing chemical pesticides with biopesticides around the reservoirs could have a positive impact. Furthermore, detailed studies are needed to investigate feeding resources, fish mortality and morbidity, and quantification of the different kinds of pollutants in both habitats. Further studies are needed to develop the standards for establishing new *ex situ* reserves, which should include criteria for location, size, topography, water quality, feeding resources, wetlands, climate, and surrounding communities. At the same time, regular surveys or investigations on the *ex situ* populations should be conducted to monitor animal health. In cetaceans, inflammatory incidences and immune function impairments are difficult to detect, therefore, cytokine markers would be helpful to monitor the health of YFPs (Fonfara et al., 2007). In terms of cytokine expression, our data suggests that the environment shapes the immune responses in YFPs. However, cytokine studies in cetacean immunotoxicology is a new field, and therefore relating the expression of certain cytokines to pollutant exposure requires further investigations. Furthermore, data on a large sample size is required to test the utility of cytokines as biomarkers.

## Data Availability Statement

All datasets generated for this study are included in the article/supplementary material.

## Ethics Statement

The animal study was reviewed and approved by the Ministry of Agriculture of the People’s Republic of China. The Research Ethic Committee of the Institute of Hydrobiology, The Chinese Academy of Sciences reviewed and approved the procedure for animals chasing, handling and blood sampling. In this study, no surgical intervention, anesthesia and euthanasia were used. The whole study strictly followed the Chinese law and ethical guidelines for wildlife.

## Author Contributions

GN conceived the study, collected and analyzed the data, and drafted the manuscript. YL analyzed the data. RM, ZM, and KW critically reviewed the manuscript. YH, JZ, and DW supervised and funded the study.

## Conflict of Interest

The authors declare that the research was conducted in the absence of any commercial or financial relationships that could be construed as a potential conflict of interest.
